# Efficacy of Electrical Cardioversion in Relation to Occurrence and Type of Functional Mitral Regurgitation in Patients with Atrial Fibrillation

**DOI:** 10.3390/jcm11082069

**Published:** 2022-04-07

**Authors:** Konrad Klocek, Katarzyna Klimek, Michał Tworek, Karolina Wrona-Kolasa, Małgorzata Cichoń, Maciej Wybraniec, Katarzyna Mizia-Stec

**Affiliations:** First Department of Cardiology, School of Medicine in Katowice, Upper-Silesian Medical Center, Medical University of Silesia, 47 Ziołowa, 40-635 Katowice, Poland; konrad.klocek23@gmail.com (K.K.); klimekkatarzyna59@gmail.com (K.K.); michal.tworek.med@gmail.com (M.T.); karolina.wrona.gcm@gmail.com (K.W.-K.); malgorzata.cichon3@gmail.com (M.C.); maciejwybraniec@gmail.com (M.W.)

**Keywords:** functional mitral regurgitation, atrial fibrillation, electrical cardioversion

## Abstract

Background: Recent studies have changed the perception of rhythm control in the treatment of atrial fibrillation (AF). Functional mitral regurgitation (fMR) can be both a cause and a consequence of AF and may influence rhythm restoration procedures. Materials and methods: A retrospective analysis included 182 consecutive patients with AF on optimal medical therapy (OMT) undergoing electrical cardioversion (CVE). Based on transthoracic echocardiography, the study group was divided into 20 (11%) patients without mitral regurgitation (MR) and 132 (82%) with fMR 77 (58%) with atrial fMR (afMR; left ventricle ejection fraction (LVEF) ≥ 50%, left atrial (LA) dilatation) and 55 (42%) and with ventricular fMR (vfMR; LVEF < 50%). Patients with severe and organic MR were excluded from the study. Results: vfMR patients had a greater incidence of kidney failure (*p* = 0.01) and coronary heart disease (*p* = 0.02); more frequent use of diuretics during hospitalization (*p* < 0.01); greater LA diameter and area (*p* < 0.01; *p* < 0.01) than afMR patients. CVE efficiency was high in all four groups (84–95%). The presence and type of fMR did not affect the efficacy of CVE (*p* = 0.2; *p* = 0.9) and did not require the use of more energy (*p* = 0.4; *p* = 0.8). The independent predictor of successful CVE was the amount of white blood cells (OR 0.74, *p* < 0.05). Conclusions: Efficacy of CVE is high among AF patients on OMT regardless of the incidence and type of fMR. Subclinical inflammation should be excluded before elective CVE because it may decrease its efficacy.

## 1. Introduction

Atrial fibrillation (AF) is the most common sustained cardiac arrhythmia in adults—in 2019, the estimated prevalence of AF among this population fluctuated between 2% and 4% [1], whereas in the next 20 years, a significant rise is expected [2,3]. AF deteriorates the patient’s quality of life [4] and increases the need for hospitalization [5]. It also creates a risk of serious complications such as stroke/systemic embolism [6], left ventricular (LV) dysfunction or heart failure (HF) [7]. AF is an independent risk factor of all-cause mortality in patients with incident AF [8].

Restoration of sinus rhythm in AF can be achieved either pharmacologically or by electrical cardioversion (CVE). There is also an option of conservative treatment and maintenance of normal ventricular rhythm with anticoagulant protection, especially in long-lasting and recurrent AF. Recent studies have changed the perception of rhythm control in the treatment of AF. According to the EAST-AFNET 4 study, maintaining sinus rhythm, even among asymptomatic patients, is associated with a better outcome [9]. CVE is the treatment of choice in patients with AF, although many factors may limit its effectiveness. One of them is mitral regurgitation (MR), which increases the risk of AF development [10,11].

It can be subdivided into two major categories: primary/organic MR and secondary/functional MR (fMR) [12].

Primary or organic MR is distinguished by the presence of structural disorder of mitral valve apparatus. However, the pathophysiology of fMR is more complex. It can be further subdivided into atrial (afMR) and ventricular (vfMR) depending on the cause of the regurgitation. The characteristic of vfMR is the imbalance between increased tethering forces (for example, LV dilation or papillary muscle displacement as a result of ischemic heart disease) and decreased closing forces (reduced LV contractility and/or synchronicity) of a structurally normal valve [13]. The following echocardiographic criteria are used to diagnose vfMR: (1) systolic LV dysfunction, (2) restricted leaflet motion and tethering, (3) eccentric jet > central jet and (4) relative left atrial (LA) dilation [14].

AfMR is a result of mitral annular dilatation and insufficient leaflet remodeling due to LA dilatation, which is the consequence of LA disease—typically occurring in the context of AF and/or heart failure with preserved ejection fraction (HFpEF) [14]. Based on echocardiographic imaging, the occurrence of (1) normal systolic LV function, (2) normal leaflet motion, (3) central jet and (4) severe LA dilation suggest the diagnosis of afMR [14].

Due to their similar pathophysiology, fMR can be both a cause and a consequence of AF and may influence rhythm restoration procedures [15]. According to S. Deferm et al. further studies are needed to clarify the impact of early rhythm restoration strategies to treat afMR [14].

## 2. Aim

The main aim of the study was to assess the efficacy of CVE in relation to the occurrence and type of fMR and the therapy used in patients with AF.

## 3. Materials and Methods

The study collected data of 182 consecutive patients (aged: 68.2 ± 11.1) consisting of 67 women and 115 men, hospitalized between January 2019 and January 2020 at the First Department of Cardiology in Upper-Silesian Medical Centre in Katowice. All patients underwent the procedure of elective CVE and met both the study inclusion (persistent AF on optimal medical therapy (OMT)) and exclusion criteria. Patients with severe or organic MR, acute coronary syndrome, previous cardiac operation in history, scheduled for valve surgery, active infection and thyroid dysfunction were excluded from the study.

**Figure 1:**
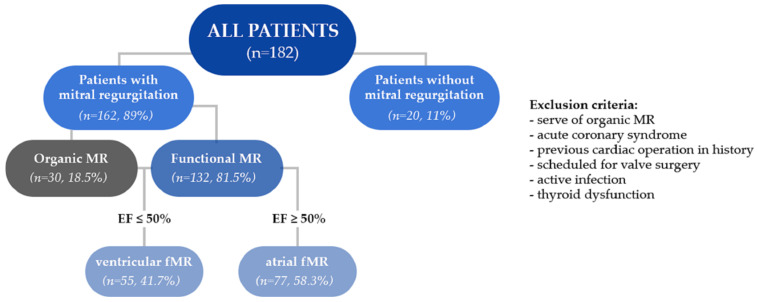
Flow chart.

Based on the results of transthoracic echocardiography (TTE) the study group have been divided into 2 major groups: 20 patients without MR (aged: 64.6 ± 16.9 years) and the second group of 132 patients with MR (aged: 68.1 ± 10 years) but without structural disorder of mitral valve or apparatus—fMR (Figure 1).

Furthermore, two subgroups were created on the basis of left ventricle ejection fraction (LVEF)—77 (58%) patients with afMR with LVEF ≥ 50% (aged: 67.7 ± 8.9) and 55 (42%) patients with “classic” vfMR (vfMR; LVEF < 50%) (aged: 69.1 ± 11.4).

### 3.1. Data Collection

A retrospective database was created from the electronic medical record and included patients’ information about demographics, chronic diseases, risk factors, TTE parameters as well as details of the CVE procedure. Analysis involved clinical characteristics, TTE parameters and efficacy of CVE—defined as restoration of sinus rhythm, amount of energy needed to perform this procedure, as well as applied pharmacological treatment.

### 3.2. Definitions

Duration of more than 7 days was accepted as a criterion for persistent AF.

Patients with fMR included those in whom primary/organic MR was excluded.

AfMR was defined as fMR in patients without LV dysfunction (with LVEF ≥ 50%), in whom the regurgitation was caused by mitral annular dilatation and insufficient leaflet remodeling due to LA dilatation in the course of its disease. Echocardiographic criteria typical for this disease (systolic LV dysfunction, restricted leaflet motion and tethering, eccentric jet > central jet and relative LA dilation) were used for classification.

In turn, vfMR was defined as fMR due to mitral valve apparatus intact and geometric displacement of the papillary muscle leaflets due to LV dysfunction and remodeling. The following echocardiographic criteria were used to diagnose vfMR: systolic LV dysfunction, restricted leaflet motion and tethering, eccentric jet > central jet and relative LA dilation. In addition, a criterion for LVEF < 50% was used.

### 3.3. CVE Procedure

In each patient, CVE was performed by an experienced physician in short-term intravenous anesthesia until sinus rhythm restoration (or further attempts to restore it have been abandoned) with an increasing amount of energy starting from 150 J using paddles.

Kidney failure was defined as an estimated Glomerular Filtration Rate (eGFR) level below 60 mL/min/1.73 m^2^.

### 3.4. Statistical Analysis

Univariate analysis was applied to both continuous and categorical variables. Categorical data were compared with chi square tests and are presented as frequencies. Continuous data with normal distribution (compared with the Student’s *t*-test) are presented as means ± standard deviation (SD). Stepwise multivariable logistic regression analyses were performed to establish the relationship between patient characteristics and efficiency of CVE. Factors taken into consideration were the occurrence of organic MR, afMR, vfMR and coronary heart disease (CHD); MR-grade (per 1 grade), MR area (cm^2^), beta-blocker usage, body mass index (BMI) (kg/m^2^), white blood count (WBC) (1000/mm^3^) and potassium level (mEq/L), LVEF (%); CHA2DS2-VASc score (per 1 point), LA diameter (LAD) (per 1 mm), LA area (LAA) (per 1 cm^2^), LV end-diastolic volume (LVEDV) (per 1 mL), LV end-systolic volume (per 1 mL) and heart rate (during AF) (per 1 bpm).

Statistical significance was considered for *p*-values < 0.05. The analysis was performed with STATISTICA 13.3 PL Software by StatSoft, Medical University of Silesia, Katowice.

## 4. Results

### 4.1. Patients without MR and with fMR

There were no statistically significant differences in baseline characteristics between patients without MR and with fMR (Table 1) regarding age, height, weight, body surface area (BSA) and BMI. Taking into consideration comorbidities and other risk factors, hypertension was more frequent in the fMR group compared to patients without MR (*p* = 0.02). Nevertheless, the occurrence of diabetes mellitus, chronic obstructive pulmonary disease (COPD), kidney failure, stroke and transient ischemic attack (TIA) in case history and CHD was similar. Moreover, there were not any statistically significant differences in the number of current smokers and patients with metabolic syndrome between those groups. 

Referring to echocardiographic parameters, a statistically significant difference was found only in LV EF (52.8% among patients without MR and 46.9% in fMR group, *p* < 0.05).

Patients without MR received pharmacotherapy in case of diuretics, antiarrhythmic drugs and β-blockers as patients with fMR.

### 4.2. Patients with afMR vs. vfMR

The comparison of groups with atrial and vfMR (Table 2) did not reveal any significant differences regarding demographic data, but the groups did not differ regardless of the frequency of hypertension, DM, COPD, stroke and TIA. However, a greater incidence of kidney failure (*p* = 0.02) and coronary heart disease (*p* = 0.02) was acknowledged among patients with vfMR. There was a significantly higher percentage of smokers in the vfMR group (*p* = 0.04).

The comparison of echocardiographic parameters between the groups (Table 3) revealed statistically significant differences in values of LA diameter (*p* < 0.01), LA area (LAA) (*p* = 0.01), left ventricular-end systolic diameter (LV ESD) (*p* < 0.01), left ventricular end-diastolic diameter (LV EDD) (*p* < 0.01), left ventricular end-diastolic volume (LV EDV) (*p* < 0.01), LV Mass (*p* < 0.01) and LV Mass Index (*p* < 0.01), which assumed greater values among patients with vfMR. In contrast, the value of LVEF was significantly lower.

The comparison of the pharmacotherapy revealed more frequent use of diuretics (*p* < 0.01) and β-blockers (*p* = 0.04) among patients with vfMR vs. afMR. The use of antiarrhythmic drugs did not differ significantly between these two groups Table 4). 

### 4.3. Efficacy of CVE in the Study Groups

The efficiency of CVE was high in the study groups, respectively, 95%—without MR; 84.1%—fMR; 84.4%—afMR; and 83.6%—vfMR. However, the comparison between groups did not reveal any statistically significant differences (Table 5.)

Furthermore, the amount of energy did not differ between groups as most of CVE were performed with energy of 150 kJ (*p* = 0.96).

Univariable analysis revealed that β-blockers (OR 0.27, *p* = 0.04) intake is a predictor of successful CVE.

Multivariable logistic regression analysis revealed that the independent predictors of successful CVE was the amount of WBC (OR 0.74, <0.05) (Table 6).

## 5. Discussion

In this study, we demonstrated that emergency CVE is effective regardless of the presence or type of MR in patients who did not require surgical intervention. The rate of successful CVEs was high, reaching 95% in patients without MR, 84% in patients with fMR, 84% with afMR and 83% with vfMR. In the context of the effectiveness of CVE, our results are similar to those presented by A.M. Fried et al. [16] that were performed on a population that did not distinguish MR.

It is important that afMR could occur in patients with AF, so treatment of AF may reduce the degree of MR. Hence, our interest in this topic was in people who, by the severity of regurgitation, were not qualified for surgery or did not have a structural mitral valve defect. In addition, it has been reported that the consequences of concomitant valvular heart disease and AF are important determinants of adverse outcomes [17]. As suggested by Zachary M. Gertz et al. [18], restoring sinus rhythm in patients with afMR improves mitral valve function. Of note, their study found that up to 80% of patients in the MR cohort had no more than mild residual MR at follow-up after the successful restoration of sinus rhythm [18]. Additionally, the restoration of sinus rhythm improves LA parameters such as dimension in long-term follow-up, as presented by A. T. Gosselink et al. [19], although worse outcomes were seen in patients with current mitral valve disease [19]. Hence, our interest in this topic is in people who, by the severity of regurgitation, were not qualified for surgery or did not have a structural mitral valve defect. However, we did not analyze patients’ degree of MR in the post-cardioversion period, which would be a very important element and worth looking at in future studies. The authors suggest that patients with afMR may benefit from early restoration of sinus rhythm through reverse anatomical and mechanical remodeling of the LA. One study addressed the efficacy of CVE for AF in patients with mitral valve disease, where it was shown that prolonged AF leads to atrophy of the LA muscles, and thus hinders CVE [20]; hence, the treatment of AF in atrial fMR prior to CVE as first-line treatment seems extremely important, and the earlier the treatment, the better the results. We have shown that the efficacy of CVE in these patients does not differ from patients with other types of MR or without this pathology, which may warrant further studies regarding the maintenance of sinus rhythm in these patients and complications associated with the CVE procedure [14]. Current guidelines do not emphasize the need to differentiate afMR from vfMR, although this study showed significant differences in echocardiographic parameters, which may indicate the validity of differentiating the two entities.

Patients in the study were on OMT for their diseases; however, they received different medications because of their multimorbidity. Special attention was given to diuretics, which are crucial in reducing LA and LV load, B-blockers and antiarrhythmic drugs, which are crucial in maintaining and converting to sinus rhythm. Medication use during hospitalization was evaluated and revealed a higher prevalence of diuretics and B-blockers in patients with vfMR compared to patients with afMR. In this study, other factors that may affect the effectiveness of CVE were not considered, such as obesity (BMI)—which causes technical problems and reduces the effectiveness of the procedure in many patients [21].

In this research, patients with mild to moderate MR, who were not eligible for cardiac surgery, were analyzed. A similar study should be performed among patients with severe MR because these patients are at high risk for surgical complications and could benefit significantly from the restoration of sinus rhythm.

The study showed that WBC count was an independent factor of CVE efficacy. Increased WBC count decreased the rate of successful CVE. It should be noted that inflammation was an exclusion criterion in our study. Based on the results, we may conclude that even a subclinical inflammation should be excluded before elective CVE because it may decrease the CVE efficacy [22,23]. B. Hunuk, in his study, suggests that elevated levels of proinflammatory markers may have proarrhythmogenic effects through cytokine induction of structural and electrical changes in the myocardium, as well as chronic sympathetic activation, which may account for the reduced efficacy of CVE [24]. In addition, according to the univariate analysis, the use of beta blockers can also affect the efficacy of this treatment. Future studies should consider the role of these parameters in CVE efficacy.

## 6. Limitations

The study presents only short-term results and did not evaluate the effect of CVE on changes in the severity of MR in a follow-up. We believe that a follow up study would have been valuable in the discussed issue, and it is currently in progress. Our other observations regarding rhythm restoration with follow-up data has been published; however, it concerned afMR and the PVI efficacy in M.Cichoń et al.’s study [25]. We have not used quantitative analysis of the degree of mitral regurgitation; however, this analysis is mainly dedicated to the severe degree of valve disease. Patients with severe and organic mitral regurgitation were excluded from the study. In the research, data from only one cardiology center were included, and a limited number of patients were analyzed. A multicenter study would be needed to obtain the most reliable results.

## 7. Conclusions

According to our research, the efficacy of CVE is high among AF patients on OMT regardless of the incidence and type of fMR. Despite the occurrence of fMR, patients should be eligible for sinus rhythm restoration. On the other hand, any form of subclinical inflammation should be excluded before elective CVE because it may decrease the CVE efficacy.

## Figures and Tables

**Table 1:** Baseline characteristics, comorbidities and risk factors comparison between groups of patients without MR and fMR.

	Without MR (*n* = 20)	fMR (*n* = 132)	*p*-Value
AGE (years)	64.6 ± (16.9)	68.1 (±10)	0.84
FEMALE (*n*; %)	5 (25%)	43 (32.6%)	0.49
BMI (kg/m^2^)	30 (±6.8)	29.9 (±4.5)	0.54
Heart rate (during AF)	92 (±25)	101 (±34)	0.42
CHA2DS2-VASc	2.8 (±1.8)	3.3 (±1.5)	0.24
Selected Laboratory Tests:			
Potassium [mEq/L]	4.4 (±0.4)	4.2 (±0.5)	0.65
WBC [1000/mm^3^]	7.8 (± 2.1)	7.1 (±1.8)	0.43
Concomitant Diseases:			
Hypertension	13 (65%)	113 (85.6%)	**0.02**
Coronary heart disease	9 (45%)	41 (31%)	0.21
Diabetes mellitus	4 (20%)	42 (31.8%)	0.28
Metabolic syndrome	5 (25%)	37 (28%)	0.78
Kidney failure	6 (30%)	38 (28.8%)	0.91
	Without MR (*n* = 20)	fMR (*n* = 132)	*p*-Value
COPD	0 (0%)	12 (9.1%)	0.5
Stroke/TIA in case history	0 (0%)	11 (8.3%)	0.92
Current smoking	1 (5%)	26 (19.7%)	0.10

**Table 2:** Baseline characteristics, comorbidities and risk factors comparison between groups of patients with afMR and vfMR.

	afMR (*n* = 77)	vfMR (*n* = 55)	*p*-Value
AGE (years)	67.5 (±8.9)	69.1 (±11.4)	0.2
FEMALE (*n*; %)	30 (38.9%)	13 (23.6%)	**0.06**
BMI (kg/m^2^)	29.9 (±5)	29.8 (±3.7)	0.77
HR (during AF)	101 (±38)	100 (±26)	0.48
CHA2DS2-VASc	2.9 (±1.6)	3.8 (±1.4)	<0.01
Selected Laboratory Tests:			
Potassium [mEq/L]	4.2 (±0.4)	4.2 (±0.5)	0.51
WBC [1000/mm^3^]	7.1 (±1.8)	7.1 (±2)	0.88
Concomitant Diseases			
Hypertension	67 (87%)	46 (83.6%)	0.58
Coronary artery disease	18 (23.4%)	23 (41.8%)	**0.02**
Diabetes mellitus	19 (24.7%)	23 (41.8%)	0.09
Metabolic syndrome	18 (23.4%)	19 (34.5%)	0.07
Kidney failure	16 (20.8%)	22 (40%)	**0.02**
	afMR (*n* = 77)	vfMR (*n* = 55)	*p*-Value
COPD	5 (6.5%)	7 (12.7%)	0.21
Stroke/TIA in case history	6 (6.5%)	3 (5.4%)	0.32
Current smoking	10 (13%)	16 (29.1%)	**0.04**

**Table 3:** Comparison of echocardiographic parameters between patients with afMR and vfMR.

	afMR (*n* = 77)	vfMR (*n* = 55)	*p*-Value
LA diameter [mm]	44.3 ( ± 4.7)	46.6 (±4.6)	**<0.01**
LA area [cm^2^]	25.7 (±6)	29 (±6.6)	**<0.01**
Posterior wall thickness [mm]	9.8 (±1.3)	10.1 (±1.3)	0.01
Septal thickness [mm]	11.9 (±2.5)	11.5 (±2.1)	0.88
LV EDD [mm]	50.6 (±5.7)	56.9 (±7.6)	**<0.01**
LV ESD [mm]	31.4 (±5.5)	42.8 (±9)	**<0.01**
LV EDV [ml]	111.7 (±25.7)	165.8 (±62.2)	**<0.01**
LVEF [%]	54.8 (±3.7)	35.9 (±9.1)	**<0.01**
LV Mass [g]	214.3 (±72.4)	252.3 (±59)	**<0.01**
LV Mass Index [g/m^2^]	107.1 (±30.1)	126.9 (±30.4)	**<0.01**
Relative wall thickness (RWT)	0.39 (±0.1)	0.36 (±0.1)	0.32
MR area [cm^2^]	6.51 (±3)	8.61 (±4.67)	0.26

**Table 4:** Comparisons of drugs used during hospitalization between groups of patients with afMR and vfMR.

	afMR (*n* = 77)	vfMR (*n* = 55)	*p*-Value
Diuretics	48 (62.3%)	48 (87.2%)	**<0.01**
Antiarrhythmics	33 (42.9%)	30 (54.5%)	0.18
β-blockers	57 (74%)	48 (87.3%)	**0.04**
Non-vitamin K antagonist oral anticoagulants (NOAC)	67 (87%)	45 (81.8%)	0.26
Vitamin K antagonist (VKA)	10 (12.9%)	10 (18.2%)	0.25
Anticoagulants—all	77 (100%)	55 (100%)	0.9

**Table 5:** Comparison of CVE results between groups of patients without MR and with fMR as well as between patients with afMR and vfMR.

	Without MR (*n* = 20)	fMR (*n* = 132)	*p*-Value
Number of successful CVE	19 (95%)	111 (84%)	0.19
	**afMR (*n* = 77)**	**vfMR (*n* = 55)**	***p*-Value**
Number of successful CVE	65 (84%)	46 (83%)	0.9

**Table 6:** Univariate analysis and multivariable logistic regression analysis of predictors of successful electrical cardioversion.

Variable	Univariable Analysis			Multivariable Logistic Regression		
	OR	95% CI	*p*	OR	95% CI	*p*
MR organic	1.60	0.45–5.73	0.47			
afMR	0.83	0.36–1.92	0.67			
vfMR	0.79	0.33–1.90	0.60			
CHD	0.48	0.21–1.13	0.09			
MR-grade [per 1 grade]	1.10	0.64–1.88	0.73			
MR area [cm^2^]	1.09	0.87–1.36	0.44			
Beta-blocker	0.27	0.08–0.94	0.04			
BMI [kg/m^2^]	0.95	0.87–1.05	0.32			
WBC [1000/mm^3^]	0.79	0.62–1.02	0.07	0.74	0.55–0.99	<0.05
Potassium [mEq/L]	1.02	0.92–1.12	0.75			
LVEF [%]	1.01	0.98–1.05	0.43			
CHA2DS2-VASc [per 1 point]	0.91	0.71–1.18	0.48			
LAD [per 1 mm]	1.02	0.94–1.12	0.64			
LAA [per 1 cm^2^]	1.02	0.95–1.10	0.59			
LVEDV [per 1 mL]	1.01	0.99–1.02	0.31			
LVESV [per 1 mL]	1.05	0.93–1.19	0.41			
Heart rate (AF) [per 1 bpm]	1.01	0.10–1.03	0.13			

## Data Availability

The data presented in this study were obtained from internal databases of First Department of Cardiology in Upper-Silesian Medical Centre in Katowice, which are restricted.
